# The Effects of Fine Particulate Air Pollution on Daily Mortality: A Case-Crossover Study in a Subtropical City, Taipei, Taiwan

**DOI:** 10.3390/ijerph110505081

**Published:** 2014-05-12

**Authors:** Shang-Shyue Tsai, Chih-Ching Chang, Saou-Hsing Liou, Chun-Yuh Yang

**Affiliations:** 1 Department of Healthcare Administration, I-Shou University, Kaohsiung 824, Taiwan; E-Mail: sionghak@isu.edu.tw; 2 Department of Environmental and Occupational Health, National Cheng Kung University, Tainan 701, Taiwan; E-Mail: chang3@mail.ncku.edu.tw; 3 Division of Environmental Health and Occupational Medicine, National Health Research Institute, Miaoli 350, Taiwan; E-Mail: shliou@nhri.org.tw; 4 Department of Public Health, College of Health Sciences, Kaohsiung Medical University, Kaohsiung 807, Taiwan

**Keywords:** fine particulate, air pollution, daily mortality, case-crossover

## Abstract

This study was undertaken to determine whether there was an association between PM_2.5_ levels and daily mortality in Taipei, Taiwan, the largest metropolitan city with a subtropical climate. Daily mortality, air pollution, and weather data for Taipei were obtained for the period from 2006–2008. The relative risk of daily mortality was estimated using a time-stratified case-crossover approach, controlling for weather variables, day of the week, seasonality, and long-term time trends. For the single pollutant model, PM_2.5_ showed association with total mortality both on warm (>23 °C) and cool days (<23 °C). There is no indication of an association between PM_2.5_ and risk of death due to respiratory diseases both on warm and cool days. PM_2.5_ had effects on the risk of death from cardiovascular diseases only on cool days. In the two-pollutant models, PM_2.5_ remained effects on the risk of mortality for all cause and cardiovascular disease after the inclusion of SO_2_ and O_3_ both on warm and cool days. This study provides evidence that short-term exposure to PM_2.5_ increased the risk of death for all cause and cardiovascular disease.

## 1. Introduction

Over the past decade, many epidemiologic studies have demonstrated positive associations between ambient levels of airborne particulate matter (PM, generally measured as PM with an aerodynamic diameter ≤ 10 µm [PM_10_]) and daily mortality [1,2,3,4,5,6,7] and hospital admissions or emergency room (ER) visits for cardiovascular and respiratory morbidity [8,9,10,11,12]. The evidence on adverse effects of PM air pollution on public health has led to more stringent standards for levels of PM in outdoor air in the USA and in other countries [13,14].

While previous studies have primarily used PM_10_ as an exposure indicator, fine particles (defined as PM with an aerodynamic diameter less than 2.5 µm; PM_2.5_) have become a greater health and regulatory concern due to epidemiologic studies suggesting that PM_2.5_ might exert greater toxicity than larger particles [15,16,17,18]. It is now generally accepted that PM_2.5_ are more harmful for health than larger particles (PM_10_) because PM_2.5_ can be inhaled more deeply into the lungs and offer a greater surface area and hence potentially larger concentrations of adsorbed or condensed toxic air pollutants per unit mass [19,20]. It is for this reason that the World Health Organization (WHO) recommends using PM_2.5_ rather than PM_10_ concentrations as indicators of air quality [7].

Relatively few epidemiologic studies have been undertaken which address the association between PM_2.5_ and rate of mortality because only few cities have monitored PM_2.5_ [17,21,22,23,24,25,26,27,28,29,30,31,32,33,34,35,36,37]. Because these studies were conducted primarily in American and European cities [17,25,26,28,29,32,33,37], the findings may not be applicable to Asian countries such as Taiwan, where characteristics of study context may be different, such as levels of PM_2.5_, population sensitivity to PM_2.5_, variability in PM_2.5_ composition and toxicity [38]. In addition, the findings have been inconsistent. Thus the association between PM_2.5_ and mortality needs further study.

To our knowledge, only a few Taiwanese epidemiological studies have investigated the acute effects of PM_2.5_, which is due to the lack of suitable monitoring data [39,40,41,42,43,44]. This study was undertaken to examine the association between short-term exposure to PM_2.5_ and daily mortality among individuals residing in Taipei city, the largest metropolitan city in Taiwan, over a 3 year period from 2006–2008, using a case-crossover design.

## 2. Materials and Methods

### 2.1. Taipei City

This study examined daily mortality in relation to PM_2.5_ levels in Taipei for the 3-year period from 2006 through 2008. Taipei is the largest metropolitan city in Taiwan with a population of approximately 2.64 million located in northern Taiwan. The major air pollution source is automobile exhaust emission. Taipei has a subtropical climate, with an annual average temperature of 23 °C.

### 2.2. Mortality Data

Total daily deaths were obtained within the Taipei city from the Department of Health (DOH), which is in charge of the death registration system, for the period from 2006 through 2008 (daily mortality data from DOH were not available nationwide after the year of 2009). For each death, detailed demographic information, including gender, date of death, date of birth, cause of death, place of death, and residential district were recorded. Deaths due to accidents (ICD-9 codes 800–999) and deaths occurring outside of the city were excluded from the analysis. The deaths were divided into the following two groups according to the International Classification of Diseases, 9th revision (ICD-9): (1) diseases of the respiratory system (ICD-9 codes 460–519); and (2) diseases of cardiovascular systems (ICD-9 codes 390–459). 

### 2.3. PM_2.5_ and Meteorological Data

Six air quality monitoring stations were established in Taipei City by the Taiwanese Environmental Protection Administration (EPA), a central governmental agency in 1994. The monitoring stations were fully automated and routinely monitored five “criteria” pollutants including sulfur dioxide (SO_2_, by ultraviolet fluorescence); particulate matter (PM_10_, by β-ray absorption); nitrogen dioxide (NO_2_, by ultraviolet fluorescence), carbon monoxide (CO, by nondispersive infrared photometry), and ozone (O_3_, by ultraviolet photometry) levels. However, PM_2.5_ was not regularly monitored, and PM_2.5_ concentrations in Taiwan have only been measured continuously since 2006. PM_2.5_ was measured using tapered element oscillating microbalance method samplers. The availability of the monitoring network for PM_2.5_ provided an opportunity to investigate the impact of PM_2.5_ on daily mortality. For each day, hourly air pollution data were obtained for all of the monitoring stations. After calculating the hourly mean of each pollutant from the 6 stations, the 24-h average levels of these pollutants were computed. Daily information on mean temperature and mean humidity was provided by the Taipei Observatory of the Central Weather Bureau.

### 2.4. Statistics

Data were analyzed using the case-crossover technique [45,46,47]. This design is an alternative to Poisson time series regression models for studying the short-term effects attributed to air pollutants [48]. In general, the case-crossover design and the time-series approach yielded almost identical results [49,50,51].

The time-stratified approach was used for the case-crossover analysis [48]. A stratification of time into separate months was made to select referent days as the days falling on the same day of the week within the same month as the index day. Air pollution levels during the case period were compared with exposures occurring on all referent days. This time-stratified referent selection scheme minimizes bias due to non-stationarity of air pollution time-series data [52,53,54]. The results of previous studies indicated that increased mortality was associated with higher air pollutant levels on the same day or the previous two days [55]. Longer lag times have rarely been described. Thus the cumulative lag period up to two previous days (*i.e.*, the average air pollutant levels of the same and previous two days) was used. Because pollutants vary considerably by season, especially O_3_ and particles, seasonal interactions between PM_2.5_ and daily mortality have often been reported. However, previous studies were conducted mostly in countries where the climates are substantially different from that in Taipei [56,57], which has a subtropical climate with no apparent 4-season cycle. Hence in this study the potential interactions of seasonality on the effects of PM_2.5_ was not considered; but temperature was used instead. The adverse health effects of each air pollutant were examined for the “warm” days (days with a mean temperature above 23 °C) and “cool” days (days with a mean temperature below 23 °C) separately.

The associations between mortality and levels of PM_2.5_ were estimated using the odds ratio (OR) and their 95% confidence intervals (CI) which were produced using conditional logistic regression with weights equal to the number of deaths on that day. All statistical analyses were performed using the SAS package (version 9.2; SAS Institute, Inc., Cary, NC, USA). Both single-pollutant models and multi-pollutant models were fitted with a different combination of pollutants (up to two pollutants per model) to assess the stability of the effect of PM_2.5_. Exposure levels to air pollutants were entered into the models as continuous variables. Meteorological variables such as daily average temperature and humidity on the same day, which might play a confounding role, were included in the model. Inclusion of barometric pressure did not markedly change the effect estimates and therefore was not considered in the final model. OR were calculated for the interquartile difference (IQR, between the 25th and the 75th percentile) for PM_2.5_, as observed during the study period.

## 3. Results and Discussion

Table 1 shows the demographic characteristics of the study population for all deaths, and respiratory and cardiovascular deaths. The distribution of air pollution, meteorologic measurements, and daily number of deaths in Taipei during the period from 2006 to 2008 are shown in Table 2. An average of 14 persons died of nonaccidental causes each day in the city over the study period. Median PM_2.5_ during study period was 27.75 µg/m^3^ (IQR:19.79–37.02), which is far higher than the USEPA National Ambient Air Quality Standard (NAAQS) of 15/m^3^ for annual PM_2.5_.

**Table 1:** Characteristics of the study population.

Characteristics		Total Deaths	Respiratory Deaths	Cardiovascular Disease Deaths
Gender	Male	9,045 (57.8%)	1,122 (67.8%)	2,539 (59.0%)
	Female	6,617 (42.2%)	534 (32.2%)	1,768 (41.0%)
Age		74.5 ± 15.4	80.6 ± 12.5	76.0 ± 13.9

The Pearson’s correlation coefficients among the air pollutants are presented in Table 3. There was a certain degree of correlation among the pollutants, especially between PM_2.5_ and PM_10_ (r = 0.91), NO_2_ and CO (r = 0.88), and between SO_2_ and both PM_10_ (r = 0.63) and PM_2.5_ (r = 0.61).

Table 4 shows the effect estimates of PM_2.5_ on daily mortality in single-pollutant models and two-pollutant models. For the single pollutant model (without adjustment for other pollutants), PM_2.5_ showed association with total mortality both on warm and cool days, with an IQR increase associated with a 7% (95% CI = 3%–11%) and 6% (95% CI = 3%–10%) rise in number of total deaths, respectively. There is no indication of an association between PM_2.5_ and total number of death due to respiratory diseases both on warm and cool days. PM_2.5_ had effects on the risk of death from cardiovascular diseases only on cool days (7% increase for each IQR range change; 95% CI = 1%–15%).

**Table 2:** Mortality counts, weather, and air pollutant concentrations in Taipei, Taiwan, 2006–2008.

Parameter ^a^	Min	25%	50%	75%	Max	Mean
PM_10_ (µg/m^3^)	15.33	36.26	48.01	62.28	205.35	52.07
PM_2.5_ (µg/m^3^)	8.25	19.79	27.75	37.02	117.72	30.65
SO_2_ (ppb)	1.12	3.07	4.05	5.35	11.14	4.32
NO_2_ (ppb)	3.73	20.66	24.53	29.55	55.51	25.37
CO (ppm)	0.15	0.53	0.66	0.82	1.73	0.70
O_3_ (ppb)	5.31	18.28	24.38	30.42	70.89	24.83
Temperature (°C)	9.35	19.67	24.32	28.27	32.78	23.74
Humidity (%)	47.92	66.95	73.21	79.28	94.19	73.02
Total deaths per day	2	11	14	17	32	14.29
Respiratory deaths	0	1	1	2	7	1.51
Cardiovascular disease death	0	2	4	5	11	3.93

Note: Abbreviation: Min, minimum value; Max, maximum value; ^a^ 24-h average.

**Table 3:** Correlation coefficients among air pollutants.

Variable	PM_10_	PM_2.5_	SO_2_	NO_2_	CO	O_3_
PM_10_	1.00	0.91	0.63	0.47	0.45	0.40
PM_2.5_	-	1.00	0.61	0.52	0.51	0.36
SO_2_	-	-	1.00	0.49	0.45	0.13
NO_2_	-	-	-	1.00	0.88	−0.03
CO	-	-	-	-	1.00	−0.19
O_3_	-	-	-	-	-	1.00

**Table 4:** Adjusted odds ratios (AORs) and 95% confidence intervals (CIs) for daily mortality for each interquartile range increase of PM_2.5_ in Taipei, Taiwan, 2006–2008, stratified by temperature.

	Total Deaths	Respiratory Disease	Cardiovascular Disease
	AOR (95% CI) ^a^	AOR (95% CI) ^a^	AOR (95% CI) ^a^
≥23 °C (617 days)			
Without adjustment ^b^	1.07 (1.03–1.11)	1.08 (0.96–1.21)	1.07 (0.99–1.15)
Adjusted for SO_2_	1.07 (1.02–1.12)	1.07 (0.93–1.24)	1.06 (0.97–1.16)
Adjusted for NO_2_	1.01 (0.96–1.06)	1.03 (0.87–1.21)	1.01 (0.91–1.11)
Adjusted for CO	0.99 (0.94–1.04)	0.99 (0.85–1.16)	0.99 (0.90–1.10)
Adjusted for O_3_	1.09 (1.04–1.14)	1.08 (0.94–1.25)	1.09 (0.99–1.19)
<23 °C (479 days)			
Without adjustment ^b^	1.06 (1.03–1.10)	1.04 (0.92–1.17)	1.07 (1.01–1.15)
Adjusted for SO_2_	1.13 (1.07–1.18)	1.10 (0.94–1.29)	1.14 (1.04–1.26)
Adjusted for NO_2_	0.99 (0.95–1.04)	0.99 (0.86–1.13)	1.01 (0.94–1.10)
Adjusted for CO	1.02 (0.98–1.07)	1.03 (0.88–1.19)	1.04 (0.95–1.13)
Adjusted for O_3_	1.07 (1.03–1.11)	1.04 (0.93–1.17)	1.08 (1.01–1.15)

Notes: ^a^ Calculated for an interquartile range increases of PM_2.5_ (17.23 µg/m^3^) and adjusted for temperature and humidity; ^b^ Single pollutant model.

In two-pollutant models, PM_2.5_ remained effects on total mortality after the inclusion of SO_2_ and O_3_ both on warm and cool days. We observed no associations between PM_2.5_ and daily mortality from respiratory diseases both on warm and cool days. For daily mortality from cardiovascular diseases, the effect of PM_2.5_ remained when SO_2_ or O_3_ was added in the regression model on cool days.

This study is one of the few that investigated the association between exposure to PM_2.5_ and daily mortality in Asia. Consistent with other studies in China [24,34,35,36], our study found association between PM_2.5_ and the risk of mortality for all cause and cardiovascular disease after inclusion of SO_2_ or O_3_ in Taipei on cool days. The observed effects of PM_2.5_ were not maintained in the presence of NO_2_ or CO. It is possible that the effect of PM_2.5_ might have been masked by those of NO_2_ and CO.

Studies conducted in US cities demonstrated significant effects of PM_2.5_ with increased mortality for all and specific causes [17,22,25,27,28]. Contrary to the large amount of US studies, several European studies failed to indicate significant association between PM_2.5_ and all cause or cause specific mortality [26,29,30,31,33]. However, with the exception of the study conducted in UK, these studies included relatively small populations (less than one million) [26,29,33] and/or short study periods (one year) [31]. In contrast, studies conducted in Barcelona (Spain) [23], Madrid (Spain) [32] and The Netherlands [37], significant associations between PM_2.5_ and daily mortality were observed.

We found a 3.48% (which corresponds to 6% increase per IQR increment) and 4.06% (which corresponds to 7% increase per IQR increment) increase of all-cause mortality and cardiovascular disease mortality per 10 µg/m^3^ increment in the 3 day moving average (lag 2) concentrations of PM_2.5_ during the cool days, respectively. The magnitude of PM_2.5_ effect estimates reported in our study were generally larger than those reported in the literatures. Ostro *et al.* found a 1.6% increase in cardiovascular mortality for an IQR increase of 14.6 µg/m^3^ for PM_2.5_ exposure [22]. Franklin *et al.* showed an association between PM_2.5_ and all-cause mortality of 1.12% and stroke mortality of 1.36% increase, per 10 µg/m^3^ of PM_2.5_ in 27 US cities [27]. Dominici *et al.* found a PM_2.5_ effect of 0.29% per 10 µg/m^3^ increase for all-cause mortality [25]. A multi-city analysis in 112 US cities showed that 10 µg/m^3^ increment of PM_2.5_ was associated with 1% increase of total mortality, 0.9% increase of cardiovascular mortality, and 1.7% increase of respiratory mortality [17]. The increase in all cause mortality associated with a 10 µg/m^3^ increase in PM_2.5_ ranged from 1.4% in Barcelona and 0.8% in The Netherlands [23,37]. Although study methods might have influenced the analysis results, disparities in findings between ours and Western populations provide evidence of the differential toxicity of PM_2.5_ with different components across locations and on various health outcomes [36]. The basis for differences in these studies is not known. One potential explanation for this discrepancy is that most published studies only demonstrated straightforward pooled effect estimates (lack of effect estimates stratified by season). This may conceal inherent differences between different climates and air pollution mixtures. Further, variations in seasonal and regional effect estimates may in part result from differences in the chemical composition of PM_2.5_ [38,58]. Nevertheless, the seasonal pattern of air pollution health effects needs to be further investigated.

In our study, associations between PM_2.5_ and the risk of death for cardiovascular diseases were observed only on cool days. The observed seasonal variation in effect estimates may be explained by variation in exposure patterns. It could be related to the subtropical climate, with very warm summers and mild winters in Taipei. The mean daily temperature rarely falls below 15 °C. People in Taipei therefore are more likely to go outdoors and open the windows in the cool season than in warm season (higher exposure); thus, monitored PM_2.5_ concentrations may be closer to personal exposure to PM_2.5_ in the cool than warm season (interaction between air pollution and temperature and better exposure assessment). This fact may attenuate the PM_2.5_-induced effect in the warm season. On the other hand, seasonal differences in air pollution mixture may also affect the effect estimates.

Some pathophysiological hypotheses may be inferred to explain the mechanisms underlying PM_2.5_-induced damage to the cardiovascular system. PM_2.5_ have been postulated as the effective toxic fraction of PM, because they promote and maintain oxidative stress both at the respiratory level (the entry system) and at the systemic level where oxidative stress induces inflammatory reactions [59,60]. Cardiovascular effects may reflect neurogenic and inflammatory processes [61]. Mechanisms for the potential effects of PM_2.5_ on cardiovascular disease may include changes in blood coagulability [62], increased circulating markers of inflammation [61,63,64], and alterations in autonomic nervous system control of the heart [2,65,66,67]. These results suggest altered hemodynamics that may lead to an increased risk of cardiovascular events.

Air pollution has consistently been associated with increased hospital admissions and daily mortality in cities throughout the world. Recent studies suggest that the increase is due primarily to PM_2.5_ [16,68]. Major PM_2.5_ components vary by region and by season, but typically include ammonium sulfate and nitrate, elemental carbon, carbonaceous species, carbonates, metals, and water [68,69]. Despite considerable research, the relative toxicity of different constituents of PM_2.5_ remains unclear but likely varies [68].

The origin of chemical pollutants in an urban atmosphere is known to be principally due to road traffic [70]. PM_2.5_ concentrations possess a less important natural component than PM_10_ concentrations. This smaller natural component makes PM_2.5_ a more reliable indicator than PM_10_ for measuring anthropic activity in a large city [71].

The case-crossover study design was proposed by Maclure to study the influence of transient, intermittent exposures on the subsequent risk of rare acute-onset events in close temporal proximity to exposure [45]. This design offers the ability to control many confounders by design rather than by statistical modelling. This design is an adaptation of the case-control study in which each case serves as his or her own referent. Therefore time-invariant subject-specific variables such as gender, age, underlying chronic disease, or other individual-level characteristics do not act as confounders. In addition, time-stratified approach [48] was found to be effective in controlling for seasonality, time trends, and chronic and slowly varying potential confounders [37,52,54]. In general, the case-crossover design and the general additive model (GAM) approach, which has been the analytic method of choice for studying short-term adverse effects of air pollution since 1990 [72], produced almost identical results [49,50,51].

For a factor to confound the relationship between PM_2.5_ levels and daily mortality it needs to be correlated with both variables. It is unlikely that smoking and other indoor pollutants confound the present association since day to day variations in indoor emissions, including smoking may not be correlated with PM_2.5_ air pollution.

Our study has several limitations. First, PM_2.5_ levels were assigned from fixed, outdoor monitoring stations to individuals to estimate exposure (assuming that exposure was homogeneous over all the studied area). Measurement errors resulting from differences between the population average exposure and ambient PM_2.5_ levels are not avoidable. However, this kind of measurement error will result in nondifferential misclassification. However, this exposure misclassification is likely to cause a bias toward the null and lead to underestimate of pollutant effects [55,73]. Second, our study population is homogenous in terms of race compared with populations in other cities and this study was conducted in a subtropical city. These facts may restrict somewhat the generalizability of these findings to other locations with different meteorological and racial characteristics. Third, behavior such as air conditioning usage or time spent outdoors may affect personal exposures. This might affect the magnitude of the observed associations compared with other geographical locations. Fourth, influenza epidemics and pneumonia morbidity may also be significant determinants of respiratory mortality. We were unable to control for these two potential confounding variables due to the lack of available information.

## 4. Conclusions

In summary, this study provided evidence of associations between short-term exposure to PM_2.5_ and increased risk of death for all cause and cardiovascular disease. The ecological design of the study precludes the inference of cause and effect. However, these findings reinforce the possible role of PM_2.5_ on adverse health effects.
